# Diabetes Management Based on the Phenotype and Stage of the Disease: An Expert Proposal from the AGORA Diabetes Collaborative Group

**DOI:** 10.3390/jcm13164839

**Published:** 2024-08-16

**Authors:** Fernando Gómez-Peralta, Pedro J. Pinés-Corrales, Estefanía Santos, Martín Cuesta, Olga González-Albarrán, Sharona Azriel, on behalf the AGORA Diabetes Collaborative Group

**Affiliations:** 1 Endocrinology and Nutrition Unit, Hospital General de Segovia, 40003 Segovia, Spain; 2 Endocrinology and Nutrition Service, Complejo Hospitalario Universitario de Albacete, 02008 Albacete, Spain; ppines77@hotmail.com; 3 Endocrinology and Nutrition Service, Complejo Hospitalario de Burgos, 09006 Burgos, Spain; esantos@alumni.unav.es; 4 Endocrinology and Nutrition Service, Hospital Clínico San Carlos, 28040 Madrid, Spain; cuestamartintutor@gmail.com; 5 Endocrinology and Nutrition Service, Hospital Gregorio Marañón, 28007 Madrid, Spain; olgagonzalezalbarran@gmail.com; 6 Endocrinology and Nutrition Service, Hospital Universitario Infanta Sofía, 28702 San Sebastián De Los Reyes, Spain; sharona.azriel@gmail.com

**Keywords:** diabetes, precision medicine, staging, diabetes complications, diabetes/disease-modifying drugs, insulin resistance, insulin deficiency

## Abstract

Diabetes is a complex and rapidly growing disease with heterogeneous clinical presentations. Recent advances in molecular and genetic technologies have led to the identification of various subtypes of diabetes. These advancements offer the potential for a more precise, individualized approach to treatment, known as precision medicine. Recognizing high-risk phenotypes and intervening early and intensively is crucial. A staging system for type 1 diabetes has been proposed and accepted globally. In this article, we will explore the different methods for categorizing and classifying type 2 diabetes (T2D) based on clinical characteristics, progression patterns, risk of complications, and the use of molecular techniques for patient grouping. We, as a team of experts, will also present an easy-to-follow treatment plan and guidance for non-specialists, particularly primary care physicians, that integrates the classification and staging of diabetes. This will help ensure that the most suitable therapy is applied to the different types of T2D at each stage of the disease’s progression.

## 1. Introduction

Diabetes is one of the most rapidly growing diseases worldwide. Traditional treatment strategies have been unable to halt disease progression or prevent the development of chronic complications. One explanation for these shortcomings is that the diagnosis of diabetes has traditionally been based on the presence of hyperglycemia. However, the disease is heterogeneous in clinical presentation and progression, with the increase in glucose being a late phenomenon [1]. Even when individuals with diabetes maintain similar levels of glycemic control, the development of complications can still vary widely [2]. This indicates that other factors beyond glycemic control may play a significant role in the progression of complications in people with diabetes [3]. Moreover, although the distinction between the two main types of diabetes, type 1 (T1D) and type 2 (T2D), has historically been based on age at onset, degree of loss of β-cell function, degree of insulin resistance, presence of diabetes-associated autoantibodies, and need for insulin treatment, none of these characteristics clearly distinguish one type of diabetes from another, nor do they account for the full spectrum of diabetes phenotypes [4].

In recent decades, advances in molecular and genetic technologies have allowed us to identify an increasing number of subtypes of diabetes. Efforts have been made to combine clinical, pathophysiological, metabolomic, and genetic features to more precisely define diabetes subgroups [5,6,7,8]. This concept of viewing T2D as a set of different phenotypes offers the appeal of a fine-tuned therapeutic approach on the path to precision medicine [9]. This approach can help define follow-up strategies and the intensity of therapy for each patient, depending on the phenotype and stage of the disease.

Moreover, the natural progression of prediabetes, T1D, and T2D is a slow process that often takes several decades to progress from the early stages to advanced co-morbid states. Unfortunately, organ damage accumulates right from the beginning, and there is limited scope for improvement in the established complications as the patient ages. The current diagnosis and definition for both prediabetes and diabetes are mainly based on glycemic criteria. It translates into two main problems. Prediabetes is associated with an increased risk of all-cause mortality and cardiovascular disease in the general population [10]. Recent and sound evidence from the UK biobank showed that subjects with prediabetes plus other specific cardiovascular (CV) risk factors (“high-risk prediabetes”) had similar absolute risks as did those with T2D [11]. Therefore, considering this population only as a category of risk for future “real” diabetes and subsidiary exclusively for lifestyle measures could be procrastination to avoid both the progression of diabetes and its related CV disease. The second challenge is that ‘prediabetes’ detection exclusively by glycemic criteria, such as impaired glucose tolerance (IGT) criteria, identifies a vast population (over 55 million in the US or 36 in Europe), impossible to manage intensively, and, probably, with an extremally heterogeneous risk for diabetes and disease progression [12]. Therefore, identifying subjects with high-risk phenotypes and intervening early and intensively is a strategic need.

A staging system for T1D has been proposed that is globally accepted and can be used as a model for other diabetes phenotypes [13]. In addition to staging individuals with autoimmune diabetes, they successfully argued against using the “type 1 prediabetes” term, correctly indicating that autoimmunity is the disease itself and hyperglycemia is a subsequent epiphenomenon. Interestingly, an updated and broad view of diabetes depicts a wide overlap between different phenotypes, including autoimmunity and insulin resistance, which are critical drivers in several diabetes phenotypes previously included under the T1D, T2D, or even type 1 + 2 diabetes terms [14].

Most routine diabetes care, especially for people with T2D, occurs in primary care settings, which are often overburdened and resource-constrained. The availability of concise protocols and guidelines for dealing with different patient profiles makes their work easier and can optimize disease management. In this article, we review the various approaches to the staging and classification of T2D based on clinical features, patterns of progression, and risk of complications, as well as the contribution of molecular techniques to patient clustering. As a collaborative group of experts, we also propose an easy-to-use management algorithm and guidance for non-specialists, particularly primary care physicians, integrating diabetes phenotyping and staging to apply the most appropriate therapy to the different phenotypes of what is currently called T2D at each point in the natural history of the disease.

## 2. Update on (Type 2) Diabetes Classification

The earliest attempt to classify people with non-insulin-dependent diabetes mellitus (later called T2D) was based on the presence of a single clinical feature: obesity [15]. Following the publication of the United Kingdom Prospective Diabetes Study (UKPDS) [16,17], the classification based on the presence of obesity was abandoned in favor of an approach focused on metformin therapy and the characteristics of the pharmacological treatment selected to achieve the glycemic goal of HbA1c < 7% [18]. However, these old T2D classification models have clear limitations in accounting for disease heterogeneity.

Correctly classifying individuals with T2D is the first step in implementing precision medicine beyond the achievement of glycemic control [9]. Cluster analysis based on pathophysiological and omics data (genomics, proteomics, metabolomics, etc.) has been used to identify and characterize subtypes of T2D [6,7,19,20]. In particular, Udler et al. [6], using data from a genome-wide association study (GWAS), identified five robust clusters of genetic loci-trait associations that likely represent mechanistic pathways causing T2D. Two clusters were related to reduced β-cell function, distinct in proinsulin levels, whereas the other three clusters were related to insulin resistance, namely obesity-mediated, “lipodystrophy-like” fat distribution, and disrupted liver lipid metabolism. Moreover, the authors identified cluster genetic risk scores associated with different clinical outcomes (e.g., coronary artery disease, stroke, and hypertension).

The primary drawback of the sub-classification approaches based on genetics is that they are not applicable to routine clinical practice. Conversely, using clinical parameters at the time of diagnosis to classify diabetes phenotypes may provide insights into the pathophysiology of the disease by considering the interplay between genetic factors and environmental influences.

In 2018, Ahlqvist et al. performed a cluster analysis of patients with recently diagnosed diabetes (*n* = 8980) that were grouped by phenotypic similarity according to six clinical parameters: presence of glutamic acid decarboxylase (GAD) autoantibodies, age at diagnosis, glycated hemoglobin (HbA1c), body mass index (BMI), and homeostatic model assessment estimates of insulin secretion capacity (HOMA2-B) and insulin resistance (HOMA2-IR) [5]. The clustering identified five different subtypes of diabetes, which were named based on their most defining characteristics.

Severe autoimmune diabetes (SAID) was defined as GAD-positive and therefore included all individuals with T1D and latent autoimmune diabetes in adults (LADA). This group had a low insulin secretion capacity, a relatively low BMI, and poor metabolic control (high HbA1c). The average age at diagnosis was around 53 years old.

Individuals in the severe insulin-deficient diabetes (SIDD) group were GAD-negative but had similar characteristics to those in the SAID group, both with a higher risk of diabetic retinopathy. The average age at diagnosis was around 60 years old.

Severe insulin-resistant diabetes (SIRD) is characterized by obesity, severe insulin resistance, high insulin secretion, late onset, relatively low HbA1c levels, and a markedly increased risk of nephropathy. The average age at diagnosis was around 68 years old.

Mild obesity-related diabetes (MOD) and mild age-related diabetes (MARD) subtypes are distinguished by early onset (average age at diagnosis 49) in the context of obesity and late-onset (average age at diagnosis 69), respectively. Both subtypes exhibit a less progressive disease [5]. This classification of adult-onset diabetes has been consistently replicated in several independent cohorts of different ethnicities [21].

**Figure 1:**
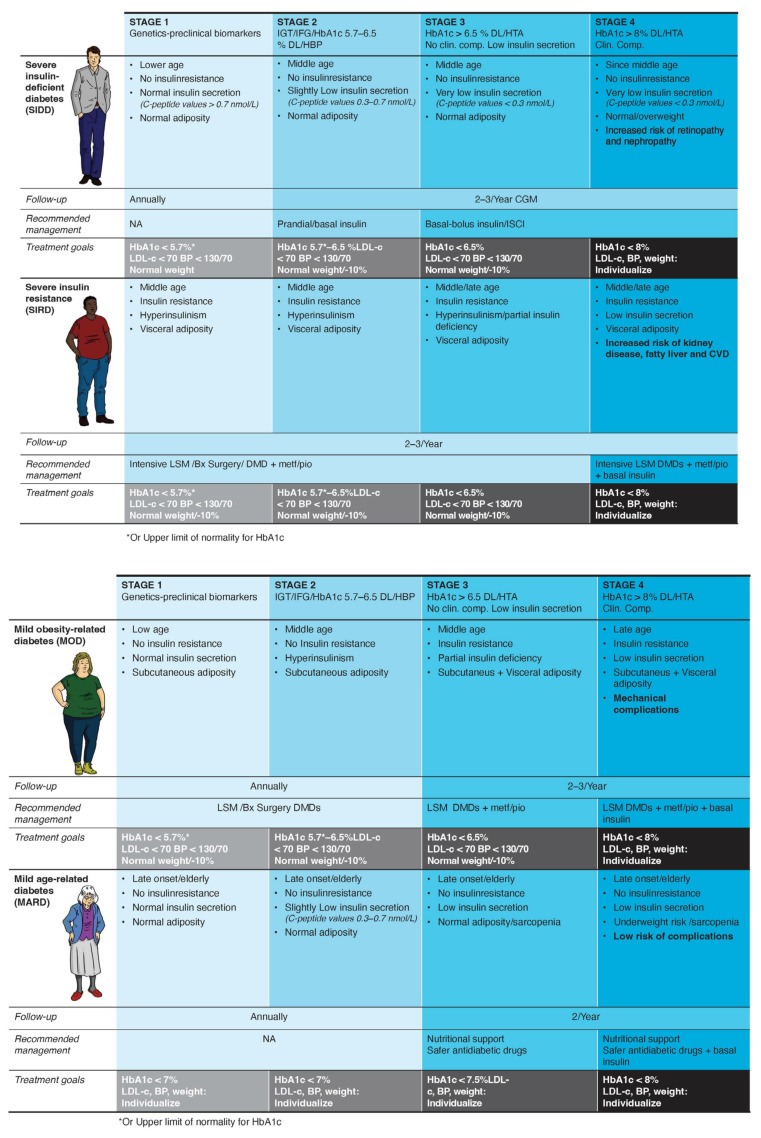
Therapeutic algorithm according to the clinical phenotype and disease stage. Abbreviations: BP, blood pressure; Bx, bariatric; CGM, continuous glucose monitoring; Clin. comp., clinical complications; CVD, cardiovascular diseases; DL, dyslipidemia; DMD, diabetes-modifying drug; IGT, impaired glucose tolerance; IFG, impaired fasting glucose; HBP, high blood pressure; ISCI, continuous insulin infusion; LDL-c, low-density lipoprotein cholesterol; LSM, lifestyle modification; Metf, metformin; Pio, pioglitazone.

An adaptation of Ahlqvist’s phenotypes for clinical use is described in Figure 1.

Phenotypic and genotypic information can help to clarify the various biological mechanisms that contribute to the development of dysglycemia in a specific individual. This approach can identify patients who are at the greatest risk of disease progression or specific complications, as well as those who are most likely to benefit from specific treatments [22,23].

One important issue commonly found in the literature on diabetes classification should be considered. Shifts from one phenotype to another over time have been described [24,25]. Intensive lifestyle or pharmacological modifications and, particularly, bariatric surgery have the potential to alter the phenotype by fostering weight loss or improving insulin resistance. Therefore, a subject can be classified into another phenotype or even reach the resolution of glycemic diabetes criteria over time [26].

## 3. Update on (Type 2) Diabetes Staging

Despite the pathophysiological complexity of T2D, its diagnosis represents the final stage of a more general disorder with insulin resistance as the central element [27]. Overt diabetes is often preceded by abnormalities in glucose homeostasis such as impaired fasting glucose and impaired glucose tolerance due to insulin resistance in the liver and muscle, respectively, which are gradual and heterogeneous processes [28]. The American Diabetes Association (ADA) does not define stages of T2D as it does for T1D. However, it does define stages prior to the diagnosis of T2D in situations related to the presence of insulin resistance (prediabetes) [1]. Some authors have developed staging proposals for T2D that focus on the presence of mild or severe complications and insulin deficiency [29]. Other proposals have used the TNM cancer staging as a template for a T2D staging system based on macrovascular events, microvascular complications, estimated glomerular filtration rate (GFR), and hemoglobin A1C to determine disease stages and substages [30].

The most workable approach for the practical staging of T2D and its management was developed by the American Association of Clinical Endocrinologists (AACE) in 2018 [31]. The proposed Stage 1 to 4 system had an exact analogy to the stages defined for T1D, representing different phases of the continuous spectrum of what they called dysglycemia-based chronic disease (DBCD). In this framework, stage 1 represents “insulin resistance”, stage 2 “prediabetes”, stage 3 “type 2 diabetes”, and stage 4 “vascular complications”. Specific prevention modalities (primordial, primary, secondary, and tertiary) are assigned to each dysglycemic stage. Avoiding overmedicalization (quaternary prevention) is implemented across stages by promoting healthy behaviors and lifestyles [31]. Importantly, the DBCD model addressed the debate surrounding insulin resistance and the diagnosis of prediabetes by including them as specific disease stages of the dysglycemia continuum, reinforcing the principle that early preventive measures can reduce the burden of cardiometabolic disease [32].

A staging system for clinical use in every diabetes phenotype is depicted in Figure 1.

## 4. Proposal of Classification and Therapeutic Algorithm

One major limitation of current clinical guidelines is the lack of guidance on how diabetes evolves over time. Additionally, the criteria for choosing between different medications do not take into account the various underlying physiological factors identified by precision medicine-based classifications. Finally, a straightforward integration of individualized recommendations for both treatment and goal-setting based on phenotyping and staging would be very beneficial for healthcare providers who frequently treat individuals with diabetes.

The primary aim of this proposal (Figure 1) is to provide a helpful and scientifically sound guide for implementing precision medicine and staging in diabetes care within primary care settings. The reasons for choosing the phenotypes of Ahlqvist et al. [5] are: first, it is scientific robustness; second, the clusters were replicated in 22 studies including people of diverse ancestries [33]; and third, the variables used are accessible to general practitioners or can be substituted by “proxies” generally available. Particularly, it does not need genetic data, which is currently not available or standardized in many health systems and populations. The Ahlqvist’ clusters were assessed close to diabetes diagnosis, allowing for adapting the phenotyping proposal to an initial subclassification of people with diabetes. Moreover, differential associations of these subtypes with clinical outcomes, including hyperglycemia progression, microvascular and macrovascular outcomes, and death, were replicated in 12 studies [33], underscoring the benefits of phenotyping. Adapting the T1D staging system to account for the evolution of the whole “diabetes syndrome” offers simplicity and intuitiveness. Notably, the boundaries between phenotypes and stages should be fluid (not rigid), because phenotypes and stages can change over time [24].

The diverse etiologic processes of dysglycemia may have an impact on the therapeutic effects of antidiabetic agents [34]. Moreover, identifying diabetes syndrome at stage 1, before overt hyperglycemia is present, may alter the natural history of the disease [35]. Our algorithm allows for a better subclassification of each case according to the risk of complications and likelihood of progression, and a more informed selection of the appropriate treatment for a given phenotype. The autoimmune subgroup, consisting of patients with T1D and LADA, should be treated according to the widely accepted ADA guidelines [36]. We recommend specific glycemic (HbA1c) targets and treatment regimens at each stage for the other four phenotypes (Figure 1). All patients should be educated on healthy eating habits and physical activity goals (at least 15 min/day of resistance training) as part of their disease management.

Some features of the non-autoimmune phenotypes should be showcased:

- **Diabetes syndrome with severe insulin deficiency:** Early detection and treatment of insulin deficiency are critical to avoid microvascular complications [37]. The use of continuous glucose monitoring is strongly recommended to guide insulin requirements by providing both real-time and predictive glycemic data [38].
- **Diabetes syndrome associated with severe insulin resistance:** Early and intensive treatment is crucial for delaying progression. Abnormal adiposity distribution and function are usually accompanied by inflammation, dyslipidemia, hypertension, and fatty liver disease [39]. Thus, this subgroup is likely to benefit from enhanced monitoring and support and the early use of diabetes/disease-modifying drugs (DMDs), such as sodium glucose co-transporter type 2 inhibitors (SGLT2i) and glucagon-like peptide-1 receptor agonists (GLP-1 RA), which are prognosis-changing agents [40].
- **Mild diabetes associated with obesity:** This phenotype is characterized by subclinical hyperinsulinism. Treatment of this subset of patients primarily involves weight loss, with a potential role for GLP-1 RA in enhancing remission of the disease beyond healthy habits [41].
- **Mild age-related diabetes:** This subgroup includes older people who have the most benign course of the disease, mainly caused by age-related impaired beta-cell function. In fact, when adjusted for current age, older age at T2D diagnosis is associated with a reduced risk of mortality and microvascular and macrovascular complications [42]. Conservative management, including medical monitoring and pharmacological treatment, is advisable.

Especially in the early stages, management strategies for diabetes syndrome should include behavioral measures beyond the healthcare system, involving educational and occupational settings. It should be directed to promote self-management and responsibility. This can be achieved by following a Mediterranean diet and engaging in regular exercise with the (ideal) collaboration of the food industry, occupational health, and local authorities to facilitate adherence to healthy habits. In elderly or frail patients (mainly at stage 4), treatment regimens should be simplified and/or deintensified to reduce the burden of glycemic management and preserve the remaining quality of life [43].

## 5. Methods and Values Recommended for the Clinical Classification of Diabetes

Ahlqvist’s clusters were based on six variables (glutamate decarboxylase antibodies, age at diagnosis, BMI, HbA1c, and homoeostatic model assessment estimates of β-cell function and insulin resistance). However, the use of clinical measures routinely collected in primary care settings and the avoidance of elaborate measures are prioritized in our approach. Therefore, after excluding autoimmunity, the proposed diabetes subclassification in the remaining four subtypes is based on age, insulin secretion, insulin resistance, and adiposity as evolutionary features (Figure 1). The text below describes in more detail the proposed methods to clinically evaluate each element. HbA1c at diagnosis showed differences between the clusters described in the original Scandinavian study. However, in real-world clinical practice, the timing of diagnosis is highly variable, and we prefer to use HbA1c as one of the management goals.

- **Autoimmunity**

The experts coincide with recent recommendations to explore the presence of autoimmunity at least once in every person with diabetes [44]. The proposed screening test is an autoantibody against glutamic acid (GADA), which is the most sensitive marker.

- **Age**

The age at the onset of diabetes was different in the Ahlqvist’ clusters. However, it showed a vast overlap. Additionally, diabetes detection in a real-world setting could be very variable. Therefore, except for the mild age-related diabetes phenotype, age should not be an absolute criterion for classification.

- **Insulin secretion**

Insulin secretion and insulin resistance must be separately and continuously measured or, at least, clinically evaluated. An age-dependent reduction in beta-cell function can affect any diabetes phenotype [45], therefore substitutive insulin therapy can be needed in every person with diabetes at advanced stages. Because C-peptide is produced in equal amounts to insulin, mirroring pancreatic beta-cell function, it represents a valuable clinical biomarker of endogenous insulin secretion in patients with diabetes [46]. When available, C-peptide values can drive therapeutic decisions. A simple approach could be random C-peptide levels to define three broad categories of insulin secretion [44]:

1. C-peptide levels < 0.3 nmol/L: a multiple-insulin regimen is recommended as for T1D.
2. C-peptide values ≥ 0.3 and <0.7 nmol/L: non-insulin agents plus basal insulin should be considered.
3. C-peptide values > 0.7 nmol/L: non-insulin agents according to the specific phenotype are recommended.

However, the laboratory indices for insulin secretion and resistance (HOMAs) used in research and C-peptide require plasmatic insulin measurements, which are not always available in a primary care setting. High fasting glucose, HbA1c goals not reached under maximum non-insulin agents, young age, and a lower or reducing weight should be considered clinical signs of insulin deficiency.

- **Insulin resistance**

Although insulin resistance plays a key role as a major and common cause of multiple metabolic diseases beyond diabetes (metabolic syndrome, atherosclerosis, polycystic ovary syndrome, and nonalcoholic fatty liver disease), the accurate detection of insulin resistance in routine clinical practice remains unresolved [47]. Clinical features suggest the presence of insulin resistance, but it is not well defined when and how insulin resistance should be assessed using laboratory tests. The canonical approach to diagnosing and measuring insulin resistance should be an assessment of oral glucose tolerance with plasmatic measurements of insulin levels. Homeostatic methods with fasting glucose and insulin measurements (HOMA) are a reasonable alternative for diagnosing insulin resistance in individuals without hyperglycemia [48]. For insulin resistance assessment, there are useful surrogate measures, the triglycerides-to-HDL-cholesterol ratio (TG/HDL-C) [49], which are commonly available in routine clinical practice and are strongly recommended instead of the complex laboratory indices.

- **Adiposity**

We chose adiposity instead of BMI because it has limitations and does not consider several important factors affecting health, such as the variation in body composition (lean/fat mass) or the different distributions of fat deposits within the body [50]. Techniques such as dual-energy X-ray absorptiometry (DEXA) and computed tomography are accurate for determining adiposity but are costly and complex for regular clinical use. More accessible methods include waist circumference (WC), waist-to-hip ratio (WHR), waist-to-height ratio (WHtR), and, particularly, A Body Shape Index (ABSI) [51]. ABSI, based on normalizing WC to BMI and height, predicts mortality independently from BMI [52] and can be useful to identify visceral and sarcopenic obesity in overweight/obese adults with T2D [53]. These are noninvasive, easy, and low-cost markers of body adiposity.

## 6. Limitations, Gaps, and Further Research

Subclassification of diabetes can be carried out in various ways, with unsupervised machine learning clustering methods often used for clinical, genomic, or other omic data. Developing treatment decision-support tools that prioritize routine clinical features could provide a low-cost and fair approach to precision treatment of T2D, especially in regions where access to essential diabetes medications is limited.

The quality of evidence supporting the clinical application of these subclassification strategies for T2D is moderate at best, and more diverse populations and high-quality evidence are needed. It will also be important to include trials specifically designed to test precision medicine hypotheses in the drug development pipeline. Furthermore, as most of the current precision diabetes medicine research has been conducted on people of European ancestry living in high-income settings, it is vital to broaden the scope to include other ethnic, geographic, and cultural groups.

In the near future, advanced methods using natural language processing and artificial intelligence will be useful as a strategy for “red flag” identification [45]. It is crucial to focus on adiposity/obesity, frequently the root cause of the diabetes syndrome and associated health issues, as there are now medications available to address it in addition to lifestyle and dietary modifications.

The clinical phenotypes described are not valid for the entire target population, there is a notable overlap between them, and individuals move with a certain frequency from one to another over time. The clinical criterion must finally be applied in each particular case through a natural derivation from precision medicine to individualized medicine.

## 7. Conclusions and Call to Action

Two main goals trigger this proposal. First, tailoring interventions to diabetes subtypes improves outcomes. Particularly, delays in insulin treatment in insulin-deficient cases and the use of DMDs in high-risk insulin-resistant ones should be avoided. Second, intensively apply all the pharmacological and lifestyle resources that can effectively change the complications, disability, and life expectancy prognosis at earlier stages of diabetes syndrome.

Probably, this approach needs a shift in the definition of diabetes itself, from those based mainly on glycemic criteria [54] to the view emphasizing that “type 2 diabetes is a chronic, heterogeneous, multi-factorial, and progressive disease characterized by inherited and acquired insulin resistance and qualitative and quantitative insulin secretion disturbances” [55]. Disseminating this physiopathological and comprehensive view to clinicians who are managing T2D is critical. Moreover, consistent with the Scientific Statement of JDRF, the Endocrine Society, and the American Diabetes Association for Staging Presymptomatic Type 1 Diabetes, the term ‘prediabetes’ should also be abandoned for the currently classified pre-clinical stages of every diabetes phenotype. In this regard, there is an urgent need to move the pharmacological resources from the overtreated elderly or frail individuals with diabetes to the undertreated earlier stages of high-risk diabetes phenotypes.

The stratification of patients based on their varying risk profiles and disease etiologies at the time of diagnosis presents an opportunity for precision medicine, which enables clinical resources to be prioritized for those who are at the highest risk of developing vasculopathy complications. This approach not only improves patient outcomes but also optimizes the allocation of healthcare resources.

## Data Availability

No new data were created or analyzed in this study. Data sharing is not applicable to this article.

## Appendix A

AGORA Diabetes collaborative group members: Abreu Padin, Cristina; Aguilera Hurtado, Eva; Azriel Mira, Sharona; Barajas Galindo, David Emilio; Bartual Rodrigo, Amparo; Bellido Guerrero, Diego; Blanco Carrasco, Antonio Jesus; Botana Lopez, Manuel Antonio; Brito Sanfiel, Miguel Angel; Capel Flores, Ismael; Castaño Gonzalez, Luis; Chamorro Martin, Jose Luis; Codina Marcet, Mercedes; Cuesta Hernandez, Martin; Darias Garzon, Ricardo; Dominguez Riscart, Jesus; Duran Rodriguez-Hervada, Alejandra; Enes Romero, Patricia; Galvan Diaz, Beatriz; Gimeno Orna, Jose Antonio; Gomez Peralta, Fernando; Gonzalez Albarran, Maria Olga; Gonzalez Perez De Villar, Noemi; Hernandez Garcia, Marta; Jimenez Varas, Ines; Lechuga Sancho, Alfonso Maria; Lopez De La Torre Casares, Martin; Lopez-Guzman Guzman, Antonio Jesus; Marco Martinez, Amparo; Mesa Pineda, Alex; Monreal Villanueva, Marta Maria; Moreira Rodriguez, Manuela; Ortola Buigues, Ana; Peteiro Gonzalez, Diego; Picon Cesar, Maria Jose; Pines Corrales, Pedro Jose; Pomares Gomez, Francisco Jose; Pujante Alarcon, Pedro; Riaño Galan, Isolina; Roldan Martin, Maria Belen; Ros Perez, Purificación; Ruiz De Adana Navas, Maria Soledad; Santos Mazo, Ruth Estefania; Soto Gonzalez, Alfonso.
